# Making Progress: ‘Sex Trafficking’, Sex Work, Temporality, and Im/mobility

**DOI:** 10.1177/13607804241275268

**Published:** 2024-12-17

**Authors:** Julio D’ Angelo Davies, Julia O’Connell Davidson, Ana Paula da Silva

**Affiliations:** University of Bristol, UK; University of Bristol, UK; Universidade Federal Fluminense, Campus do Gragoata, Brazil

**Keywords:** mobility, sex work, temporality, trafficking

## Abstract

Anti-trafficking discourse is built upon and reproduces a series of either/or conceptual binaries (voluntary/forced, consensual/coerced, agent/victim) which obscure the unseen, structural factors that shape the fates of men, women, and children under the economic, social, and political relations of global capitalism, as well as their experience as workers in given countries, sectors, and workplaces. Some sex worker rights activists and scholars have contested its application to prostitution by emphasising that ‘not all sex workers are trafficked’. However, this position also privileges the question of whether an individual voluntarily chose or was coerced by a third party into a given form of work. Drawing on biographical interview data with women in sex work in Brazil that shows how past, present, and hopes for the future are woven together in their sex-work trajectories, this article adds to the literature that critiques anti-trafficking discourse through a focus on temporality. It argues that by following the trajectories of sex working women’s lives over time, it is possible to better grasp both the protean nature of sex work, and the impossibility of fixing people’s participation in it as either forced or free.

## Introduction

As soon as ‘trafficking’ emerged as an issue of public and policy concern in the 1990s, critical scholars and activists started to question the assumptions about gender, race, prostitution, and mobility that underpinned it (Chapkis, 2013 [1997]; Kempadoo and Doezema, 2018 [1998]). Today, there is a substantial body of literature highlighting the ways in which ‘anti-trafficking’ discourse has encouraged and legitimated more restrictive immigration policies and tighter border controls, and/or helped advance extremely conservative moral agendas on prostitution, gender, and sexuality, and fostered repressive policies against people who sell sex (e.g. Agustín, 2008 [2007]; Bernstein, 2018; Chapkis, 2005; Cruz et al., 2019; Kempadoo, 2007; Kempadoo et al., 2005; O’Connell Davidson, 2006; Parmanand, 2019; Schaffauser, 2010; Shih, 2021; Silva and Blanchette, 2017; Walia, 2021). Within this literature, there are sex-worker rights scholars and activists whose main criticism of dominant anti-trafficking discourse is that it vastly exaggerates the prevalence of trafficking in the sex sector, not least because it wrongly assumes that female sex workers lack agency. Thus, one strand of critique stresses that ‘sexual commerce qualifies as work, involves human agency, and may be potentially empowering for workers’ (Weitzer, 2007: 215), and holds that trafficking is no more of a problem, and may even be less of problem, in the sex industry than in other economic sectors (Weitzer, 2012: 1369).

However, this response continues to work with the conceptual binaries of dominant anti-trafficking discourse, and so to accept the idea that human action (whether in migration, sex work, or other forms of work) can be neatly boxed as *either* voluntary *or* forced, and people neatly classified as *either* agents *or* victims, subjects *or* objects. As other scholars and activists have observed, insisting that ‘not all sex workers are trafficked’ repositions the majority of sex workers on the ‘free’ side of the dichotomy, rather than challenging the either/or conceptual binaries of voluntary/forced and consensual/coerced that scaffold the entire anti-trafficking paradigm (Cruz, 2018; Doezema, 1998; Kotiswaran, 2021; O’Connell Davidson, 2010; Smith and Mac, 2020 [2018]).

This article, which presents biographical interview data from research with women who sell sex in two Brazilian cities, asks whether closer attention to temporal dimensions of sex workers’ experience might help us to challenge dominant discourse on ‘sex trafficking’ without reproducing these conceptual binaries. It applies the insights of migration scholars who foreground temporality in their critiques of anti-trafficking to sex work. Migration scholars have shown how the journeys of unauthorised migrants often take place over long periods of time, sometimes years, and can include periods of forced immobility as well as time on the move (e.g. Anderson, 2013; Bastia and McGrath, 2011; Griffiths et al., 2013; Stock, 2019), and noted that such journeys cannot be neatly slotted into the administrative and conceptual categories used to distinguish between supposedly distinct forms of ‘migration’, such as forced/voluntary, trafficked/smuggled, asylum-seeking/economic, and temporary/permanent/transit (Martins and O’Connell Davidson, 2022). People’s experience of sex work also spans time, and they can move in and out of the kind of conditions described as ‘trafficking’ or ‘forced labour’, and we follow Sallie Yea (2012) in arguing that to ‘move beyond essentialist images’ (p. 56) of sex workers as either passive victims or empowered sex worker agents, it is necessary to consider both time and space.

Our interviewees narrated their participation in sex work as a strategy by which to escape situations in which they felt trapped and unable to achieve, or even make, life plans. Like participants in Mai’s (2018) research, they decided ‘to work in the sex industry in order to fulfil their mobile orientations, have a better life, and escape the forms of exploitation they meet in other jobs’ (p. xv). That decision was an active but not necessarily a positive choice, given the absence of alternative possible means of pursuing life projects. Moreover, some of our research participants had indeed experienced what dominant discourse would cast as ‘trafficking’ or ‘modern slavery’. Sex work itself was not idealised by our research participants. However, reflecting back on several years in this form of work, they did value the social and spatial mobility that it had allowed them. The article concludes that to freeze-frame the analysis of such women’s participation in sex work either at the moment of extreme exploitation or at the moment at which they exercise greater choice and control is to disregard the ambiguity and fluidity of their experience.

## Research methods

The data discussed in this article were gathered as part of a larger, European Research Council funded project that included field research in Brazil addressing questions about the pursuit and practice of freedom in and through sexual and intimate relationships. As part of this study, biographical and focus-group interviews with written consent were conducted with a purposive sample of 18 women sex workers in two cities, Rio de Janeiro and Ribeirão Preto. In Rio de Janeiro, all research participants were recruited through a sex worker collective that was created as a space for support and self-help among women who work in the sex trade in the neighbourhood of Copacabana, a site where there is strong demand for commercial sex from tourists, as well as locals. In Ribeirão Preto, they were recruited through a sex worker inclusive non-governmental organization (NGO) that has been providing care and assistance to local sex workers since the 1990s. We chose to access the sample through the collective and NGO to ensure that our research participants were in touch with organisations, activists, and services that could support them should participation in the interview leave them feeling emotionally stressed. We also chose to ensure that all our participants were aged above 18, and none had drug or alcohol dependencies. Again, this was for ethical reasons, this time to ensure that participants had full capacity to consent to participate in the research. Given the exclusion of people below 18 and with problems of addiction from the research sample, we acknowledge our data reflect the experience of a particular subset of the population of sex workers. The expenses that women incurred to travel for interviews were reimbursed, and they were financially compensated for lost working time after the interview, but payment was not offered as an inducement to participate in the research. Pseudonyms chosen by our research participants are used in this article to preserve their anonymity.

Fieldwork was undertaken by Ana Paula Silva in Rio de Janeiro between March and May 2022, and by Julio D’ Angelo Davies in Ribeirão Preto, state of São Paulo, in January 2023. The biographical and focus-group interviews lasted between 1 hour and 3 hours. In Rio de Janeiro, a total of 10 biographical interviews and two focus-group interviews were undertaken in local bars, restaurants, and hotel conference rooms in the neighbourhood of Copacabana, where the women undertake sex work. Our interviewees in Copacabana were cis women and from working class backgrounds. One had migrated to Brazil from a Caribbean country, three had migrated to Rio from other states in Brazil (Rio Grande do Sul, Minas Gerais, and Espírito Santo), while the rest were from peripheral regions of Rio de Janeiro (the Baixada Fluminense and the city’s favelas). In Ribeirão Preto, two focus-group interviews and eight biographical interviews were conducted. Interviews were conducted in the offices of the NGO that supported the women. Only one participant was originally from Ribeirão Preto. Six had migrated there from rural areas, and one commuted from a rural town to the city to work. All came from working class backgrounds, four participants were cisgender and four were trans women. So far as identifying the racialised identity of our participants is concerned, it is important to remember that race thinking in Brazil has historically differed and still differs from race thinking in the United States and the United Kingdom:

the construction of racial differences shows that for Brazilians, colour matters more than ancestry. One rarely speaks of ‘whites’ and ‘blacks’, relying rather on a colour gradient (light-skinned, tan, dark-skinned, etc.) linked not only to the degree of social distance between the interlocutor and the object, but also to the context in which they are situated. (Rezende and Lima, 2004: 761)

Nonetheless, the Brazilian census does use racial categories, asking respondents to identify as *branco*, *pardo*, and *preto*, which approximately translate as ‘white’, ‘brown’, and ‘black’, respectively (Bailey and Telles, 2006). All 18 of our research participants self-identified as *preta* or *parda*.

## Foregrounding violent exploitation

Anti-trafficking and anti-modern slavery campaigning materials and books often open with a particularly distressing example of a woman or child who fell victim to the phenomenon that they describe as ‘trafficking’. We will follow suite, and open with a situation described by Isabelle, one of our research participants in Ribeirão Preto. A woman friend of Isabelle’s, who already had experience as a sex worker, suggested they should leave their rural hometown to earn money through sex work in Ribeirão Preto. Isabelle agreed, and her friend contacted a nightclub owner who offered them work. He sent them bus tickets to Ribeirão, met them at the bus station and told them that at the nightclub, they would be provided with accommodation and meals, and that they would earn 150 *reais* for each *programme* (service or ‘trick’) they sold. He explained they would work all week, but on Saturdays the club was closed and they were free to go home. ‘For me it was great’, Isabelle said, ‘I would be able to spend the weekend at home’.

The man took them by car to his nightclub, and doubts quickly set in. Isabelle and her friend realised their earning opportunities were going to be far more limited than they had imagined. The nightclub was in a remote location, it was not buzzing with customers, and there were already several other women working there. Isabelle also learned of a further obstacle to earning. Before being allowed to take a client to a private room for a *programme*, the owner insisted that sex workers had to ensure the potential client spent at least 80 *reais* on drinks in the bar:

So you never managed to get a *program*. When the guy asked you, ‘How much is it?’, you would say, ‘It’s 150 *reais*, 30 for the room’. Then he would say, ‘Let’s go then’, then I would say ‘But you have to spend 80 *reais* on drinks at the bar’, and he would say ‘No, I won’t spend that much! Are you crazy, girl?’

Isabelle told us ‘One day passed, nothing. Two days, nothing’. The food was also bad, ‘just spaghetti with sausages, and we even had to cook it ourselves’. But they still owed the nightclub owner the money for their bus tickets. So there they were, in the middle of nowhere:

and I said, ‘we are in a bit of a jam here’ . . . without even one *real* in our pockets . . . Then I told my friend ‘I’m out of here’, and she said ‘How Isabelle? How are we going to get away?’ Because there were three pit bulls that the owner would release in the morning, there, at the edge of the pool in the house of this nightclub where we stayed. And she said ‘Isabelle, if we run, these pit bulls will tear us apart’. I told her ‘Look, didn’t he say that the club doesn’t open at weekends? Then he will leave’.

The owner had told them they could return home for the weekend if they wanted to, knowing that they had no money to pay a bus fare home. However, he also told them he would not sleep at the club on Saturday:

I said, ‘When he leaves, we will jump over this wall and leave’ . . . We dozed off a little in the bedroom, when I woke up, it was about 6am. Then I called to my friend ‘Let’s get out of here because the man is not there!’ But when we were passing through the hall, I heard someone snoring in the next room. And there were cameras everywhere, right? I said to her ‘The man didn’t leave!’ Then she started crying, ‘He did this on purpose, this man is going to kill us, he suspects that we are going to run away from here’, and I said ‘Whether he kills us or not, I’m not going to stay here!’ When we went outside, thank God the caretaker hadn’t let the dogs out yet . . . We walked to the gate, there were a couple of padlocks . . . I said to myself, ‘Now you fucked up!’ And the high walls! And I looked and said ‘I’m going to jump this wall, I’ll break my legs down there but I’m going to jump. I won’t stay here’ . . . I thought, ‘This man is going to kill us’ . . . But it must have been God, because when I looked under the gate, there was . . . an iron stick . . . When I lifted it up, the gate opened . . . Oh, but when the gate opened it seemed as if I saw God in front of me. I looked around and there were only bushes and a motel next to it, because when we arrived at night I didn’t even pay attention to where we were. And we stayed the whole time without going outside, everything locked up, right? Day and night inside. I saw a track down by the river, and I said ‘My friend, run, run!! We will get to the track and ask for a ride’

Fortunately, a car passed, the driver offered them a lift, and they made their escape.

Tamires, another research participant in Ribeirão Preto, observed that women who work in brothels (referred to as houses) in more remote rural towns are particularly vulnerable to this kind of maltreatment. She too had once found herself in a house where the Madam demanded that sex workers worked a 12-hour shift daily, cleaned the bar, and washed up for her afterwards, and locked the women in at night. She explained,

Here in Brazil, the biggest form of exploitation is this . . . setting up distant houses, withholding women’s payments, giving fines . . . invalid fines, because I have no employment relationship with the house, I cannot be fined. But this happens a lot, it has happened to me . . . and to several of my colleagues here.

Prostitution is represented as a common site of ‘modern slavery’ by anti-slavery activists (Bales, 1999; Kara, 2009) and described as ‘sexual slavery’ by radical feminists (Barry, 1984; Bindel, 2017; Jeffreys, 1997). Melissa Farley et al. (2017), for example, claims that as with relationships between the enslaved and their legal owners historically, relationships between women in prostitution and their employers and customers are ‘characterized by control of movement, control of the physical environment, psychological control, measures taken to prevent or deter escape, force, threat of force or coercion, subjection to cruel treatment and abuse, control of sexuality and forced labour’. If we focus only on the moment in time at which Isabelle and Tamires experienced the kind of force and exploitation described earlier, analogies with slavery may appear well-founded, and the kind of interventions encouraged by the anti-trafficking apparatus (‘raid and rescue’, measures to discourage or prevent women and girls from moving to work in the sex industry etc.) may seem appropriate and laudable. As with unauthorised migrants (Anderson, 2013; Griffiths et al., 2013; Stock, 2019), journeys of sex workers can present varying conditions over time and space. Temporality shifts between situations of forced immobility, as reported by Tamires and Isabelle, to periods of time on the move. Therefore, as we aim to show below, approaching our interviewees’ engagement with sex work over time, rather than viewing exploitation as a static event (Waite and Lewis, 2017) generates a rather different picture.

## Life before sex work

Our research participants live in a country ranked as the world’s sixth most unequal according to the World Bank’s widely used Gini coefficient measurement, and the most unequal country in Latin American (Degenhard, 2023). The scale of income inequality in the country is so monumental that, according to Oxfam (2023), Brazil’s six richest men hold the same wealth as the poorest 50% of the country’s whole population and the top 5% have the same proportional income as the remaining 95%. Economic inequalities in Brazil are rooted in the country’s history of slavery and colonisation, and as a result, such inequalities are racialised. Census data show that around 45% of the population identifies as white (*branco*), yet 79% of owners of large agricultural holdings are white. In 2021, just 18.6% of *branco* people, but 34.5% of *preto* (black) and 38.4% of *pardo* (brown) people were living below the poverty line (Cabral, 2022). White Brazilians earn 75% more than *preto* and *pardo* Brazilians (Capetti and Almeida, 2019). *Preto* and *pardo* people are more likely to be employed in the informal economy than are people racialised as *branco*, while white Brazilians are disproportionately present in managerial jobs (Cabral, 2022). Statistically, black and brown Brazilians are more likely than white Brazilians to fare poorly in terms of life expectancy, treatment in the public health services, access to housing, sanitation, education, homicide rates, and are also more likely to die as a result of police actions (Cabral, 2022; Gatehouse, 2012; Pimentel, 2022).

Gender inequalities intersect with inequalities of race and class. Women’s labour market participation in Brazil grew from the 1960s, but the labour market remains strongly gender segregated, with women heavily concentrated in the tertiary sector. Within this, however,

distinctions resulting from differences in social origins led in turn to differential positions for white, black, and mixed-race women . . . Having less schooling than white women and therefore fewer skills . . . Women from lower-income groups tended towards services provided in industry (e.g., outsourced janitorial work in factories) and domestic work, while middle-class women tended towards collective services (e.g., health and education) due to their higher educational levels. (Rezende and Lima, 2004: 762)

The overrepresentation of *preta* and *parda* women in unskilled service work, especially domestic work and other informal sector jobs, helps to explain the fact that they currently earn ‘approximately 44.4% of the average income of White men, who are the top occupants of the country’s wage scale’ (Pimentel, 2022). Moreover, despite extensive efforts to address the problem in law:

violence against women – especially femicide – has persisted at high levels in the past decade. Brazil consistently ranks as one of the most dangerous countries in the Americas and globally for gender-based violence . . . Brazil is also one of the most violent countries for LGBTQ+ people. According to global data collected between October 2019 and September 2020, Brazil witnessed the highest number of murders of trans and gender diverse people of any country, having reported 43 percent of the global total. (Walters, 2020)

In this context, Brazilians from poor and working class backgrounds, especially women racialised as *preta* or *parda*, face multiple challenges in their day to day lives. The structural inequalities outlined earlier position them in such a way as to make daily life precarious, and, as avenues of social mobility are so few, render it almost impossible to see a realistic course to changing that position. Such was the case for our interviewees prior to commencing sex work.

All of our Brazilian research participants had worked in other occupations before starting sex work, and several started work as children. Gisele, a research participant in Copacabana, described herself as coming ‘from a humble family’. She grew up on the outskirts of Rio de Janeiro, the eldest of three siblings in a single parent household. As a child, Gisele worked as a maid to earn money to contribute to their survival. Her education was interrupted and she did not finish high school. At 16, she left home and worked as a waitress and in other service sector jobs, but was paid very little. Life as a teenager was ‘really tough’, in her words. She was unable to afford simple things, like a deodorant and ‘basic things like a tampon . . . I didn’t have . . . where would I get it from? These are things that women must have and I had nowhere to get them from’.

Gisele was always looking for ways to extricate herself from this situation, and proactively pursued opportunities for better work. She attempted to return to education, and even managed to enrol on an engineering bachelor’s course:

I tried everything!’ . . . But it was very difficult, I couldn’t reconcile life with studying engineering, very expensive . . . even though I had sixty percent off the tuition via a scholarship, it was still too expensive.

Though other research participants had finished primary education and some had experiences with higher education, they had all laboured in precarious, low-wage jobs, often without formal employment contracts, mostly in the service sector or agriculture. Isabelle, whose terrifying experience of being confined in a nightclub was described earlier, told us that her first employment, which she remained in for 3 years, was cutting sugar cane in the fields – ‘God, it was horrible!’ She subsequently took up a job as a domestic worker, an experience she describes as extremely painful and exploitative, and had also had jobs in retail, snack bars, hotels:

I worked a lot and earned very little . . . my last job was in 2020. They hired me for six months. A salary of 1500 *reais* . . . And you work when the harvest season starts because as the contract is only for the harvest season, sometimes you can spend a whole month without a day off. Sunday to Sunday. You leave home at 7am and start working at 7:30am. When you leave work it’s 10 p.m., 11 p.m., midnight. The next day you have to be there again at 7 a.m.

Other research participants in Ribeirão Preto had also worked cutting sugar cane and/or picking oranges or coffee, work that they described as ‘suffering’ to earn barely enough to survive. In the service sector too, our interviewees generally earned well below the minimum wage, and even those who had found higher status jobs had not earned enough to make their own lives and those of their dependents materially secure. Certainly, they had all been excluded from the forms of consumption that are taken as markers of social status and command respect in contemporary Brazil. Moreover, some of our research participants had experienced sexual harassment in their workplaces.

Most of our research participants had children and/or parents and other family members who relied on them economically, including dependents who were chronically ill or suffered mental health problems. In some cases, women had had children with men who provided no financial or emotional support to them as parents, and several interviewees had experienced domestic violence in marriages or relationships they entered as teenagers or young women. Isabelle, for instance, told us that

I used to live . . . in an abusive relationship . . . If I asked my husband for money, ‘Oh, you are too lazy, this and that!’ . . . I used to be beaten. The guy would slap me in the face and I would fall on the floor. We used to fight with punches inside our home and the children seeing all that.

In other words, where anti-trafficking discourse focuses attention on prostitution as the site of violence and restraints on freedom, many of our interviewees experienced violence in their domestic and intimate relationships prior to, or alongside sex work, and all of them described having felt trapped in lives they did not choose but could see no way to escape. Again, temporality is essential to contextually comprehend sex workers’ shifting journeys. As Isabelle’s narrative shows, marriage can also be a site of forced immobility and violence depending on the time frame we choose to illustrate. Sex work promised a route out of this kind of stasis, a means to mobility and progress, as reported by many research participants.

## Making progress

Against the backcloth of the kind of jobs and intimate relationships described earlier, our Ribeirão Preto research participants narrated their movement into sex work in the city as a way of ‘evolving’ or ‘making progress in their lives’:

Leandra: In [my hometown] my life was very slow . . . I worked in the fields . . . hard work. Suffering like hell! The strongest moment was when I decided to leave and come to Ribeirão Preto. That’s when I started to make progress in my life! I was 12 when my father passed away and my mother also started working in the orange plantations because life started to get very difficult. And I had to do my own thing, right? Nobody ever gave me anything. It was prostitution that opened this door for me, this path.

Bella: [In my hometown] it was a lot of suffering. Then this . . . friend of mine . . . came [to Ribeirão Preto] with her friend who introduced prostitution to her. Then she told me ‘Let’s go there, Bella, your life will get better, everything will get better!’ And I went with her.

Bella, now aged 34, moved to the city and started sex work in 2018 at the age of 29. Later, she brought her son, mother and two nieces, who depend on her financially, to live with her in Ribeirão Preto. With her current earnings, she is able to pay 2000 *reais* monthly for her son’s treatments in a private clinic for autism and learning disabilities. Bella also buys her mother’s expensive controlled medication for her psychiatric needs.

Leandra is a trans woman in her late 30s and unlike most of our cis interviewees, does not have children and grandchildren to raise. Nonetheless, after learning about sex work through friends, including other trans women, and eventually starting a new life in Ribeirão Preto, she supports both herself and her mother. With her earnings, she has also been making national insurance contributions on her mother’s behalf in order to guarantee her mother a decent retirement, something her mother could not achieve through plantation labour:

Today my mother is going to retire, thanks to me, because I have been paying her INSS [National Insurance] for many years. One more year to go. Strictly speaking, since I started working, I pay for myself and I pay for her. So it was a help, right? Let’s say, I made the burden of my family a little lighter.

Leandra also helped in other ways. After her father passed away, council tax bills were not being paid and the family’s house was falling to pieces. Her earnings allowed her to refurbish the property and pay all the family’s debts. ‘I think that if I hadn’t gotten into prostitution we would have lost this house’, she said. Gisele, quoted earlier, explained that sex work has enabled her to achieve ambitions that would otherwise have been unattainable:

I had my breast implants . . . I finished paying my car loan . . . and I bought my house five months ago, I have just finished paying for it . . . My house is valued in a hundred thousand *reais* . . . I know this might not sound a lot, but where I come from this is a lot of money (laughs) . . . I must be the only one in my family who owns a house.

Tanya, who grew up in an extremely poor slum area of Rio de Janeiro, told us that as children, she and her two siblings would help their mother who worked as a street vendor selling candies and ‘When we were short of a lot of stuff at home, we would go down to beg for food on the streets’. Tanya began to trade sex as 16-year-old, after seeing some of her friends managing to earn enough money in this way to be able to buy new clothes and hang out in leisure venues. Now aged 21, Tanya has a child, and has experienced two domestically violent long-term relationships. She continues to sell sex, but works in Copacabana, often with tourist clients, earning relatively good money. She says she hopes to be able to study or train for a different form of employment:

I don’t want to reach my thirties and say to my son, ‘Son, I’m a call girl’. No, I don’t want that, God forbid, you know?

Nonetheless, she describes sex work as having allowed her to move forward in her life:

Before I lived like shit, nowadays I can pay a little more for rent than I could before, you know. And it gave me the chance to go somewhere, to visit good places, places that I’ve never been in my life, you know? And this is wonderful for me . . . I was able to see an improvement, understand? To dress better, to eat better, to do some things better. Not everything, I’m not going to tell you that it’s a miracle, it’s not . . . But a lot of things, I manage to do things.

In the case of another Copacabana research participant, sex work was a route to achieving a career as a teacher in the state public network in Rio de Janeiro. Naara paid for her studies from high school on, including a master’s degree, with money from sex work. She grew up in a city in the interior of Minas Gerais state, where she completed her undergraduate and master’s degrees at public universities. Although she did not have to pay tuition fees, the money she earned from sex work helped her to participate in national and international conferences which were crucial to her success in public employment competitions after completing her master’s degree. She was approved for a teaching vacancy in Rio de Janeiro. In Naara’s words,

I left the outskirts of a small town in the interior of Minas Gerais, where my family had no money, I was forced to hide my poor origin, pretend I was white, straighten my hair and lighten my skin; a place where I could at best be a maid for the local elite. Sex gave me everything. Sex is power. Power is money. In an unequal capitalist society, this is what matters. I achieved something. I have the freedom to travel where I want, drink and eat well. I got an education and opportunities because I used the power of sex. Today I am a teacher. I want to do my doctorate and – who knows – in the future to become a university professor.

In short, participation in sex work had allowed our research participants to make significant improvements in their lives, both in terms of being able to provide for themselves and their dependents, and in terms of opening up their horizons. It made it possible to live in cosmopolitan cities, and to pursue opportunities for achieving their various aspirations regarding homeownership, education, careers, and/or further migration, as well as to access consumer and symbolic goods that they consider important in a capitalist society. Eating well, getting to know good wines, travel, being able to leave Brazil, achieving a university education – these are all common aspirations in a capitalist society, but aspirations that are not easily achieved by poor, Brazilian girls and women, racialised as black or brown, who live in peripheral areas. Certainly, they would have been inaccessible to our interviewees had they remained in their old jobs in the formal and/or informal sectors of the economy, which paid a minimum wage or below. It was the possibility for this kind of mobility and for independence, not sex work itself, that our interlocuters valued above all. As Tamires put it when asked what she had been able to achieve through sex work:

It’s a utopia to think that you’re going to be rich, that you’re going to make a lot of money. When I started working, I bought my plot of land, I [bought] a car . . . I had a son. My son got sick at the time, very sick indeed. I had to sell my things to pay for his treatment. He passed away. But today I have my car, I am building my house . . . It gave me the freedom to know places, because I like to travel, I like to buy my books, right? It means I can pay my own expenses . . . Sex work gave me this freedom to come and go and not having to be tied to anyone. I am a person that likes to be alone, I don’t like anyone controlling me, I don’t like anyone counting what I spend. So it’s not a question of money, it is because I like my freedom . . . I have had relationships where I’ve had this problem of the person wanting to take charge. The money gives me the freedom . . . to come and go. I like to have this freedom of movement.

By considering Tamires’, Bella’s, and Leandra’s participation in sex work through a temporal lens rather than freezing it at a particular point in time and attempting to decipher whether at that moment it was forced or voluntary, we can better understand the limitations of efforts to divide sex workers into the categories of ‘victim’ or ‘agent’.

## Beyond the free/forced dichotomy

In radical feminist writings, prostitution appears as akin to slavery in terms of the violence and degradation it implies. Some ‘sex positive’ sex worker rights thinkers invert this logic to depict sex workers as resistant sexual subjects (Bell, 1994; Rubin, 2002), even ‘the unsung heroes of sexual liberation’ (Gallant, 2021). Our research participants’ stories fit with neither of these characterisations. They actively decided on sex work, and their experience of it was not always and irredeemably violent. But nor was it always or uniformly positive. Our interviewees were not miraculously transported into workplaces free of the discrimination experienced in other types of work – they described having to deal with racism from clients, for example. Nor were they suddenly freed from economic precarity and exploitation. Some had initially earned very little from sex work then progressed to fairly consistently making relatively good money from it. But for others, earnings from sex work still fluctuated (and many sex workers in Brazil were very heavily and negatively affected by the COVID-19 pandemic and associated lockdowns, see Martins et al., 2023). And though some described having exercised a high level of control over their own sex work throughout, others – as described at the start of this article – had experienced very serious abuse and exploitation.

Returning to Isabelle and Tamires’ stories through a concern with temporality and asking what happened next illuminates the problem with either/or thinking. As already noted, Isabelle and her friend were offered a lift by a man as they were escaping the nightclub. Once in his car, they told him the whole story of what had happened to them. He did not have time to take them all the way to the bus station in Ribeirão Preto but dropped them at a bus stop and gave them money to pay the fare into the city. He also told them about a bar where sex workers could work independently, explaining that there, they would make their own money and be free to come and go as they pleased. They followed his recommendation, and ‘when we got there, people were very welcoming to us’. Isabelle’s friend was so scared by their experience that she only did one ‘program’ in Ribeirão, used the money for her bus ticket, and left. But Isabelle made the decision to stay and continue in sex work. Likewise, Tamires, who had found herself locked into house and heavily exploited, told us that she had said to the Madam who employed her:

‘This is false imprisonment, you are aware of that, right? . . . this is private restraint, okay? It’s a criminal offence.’ . . . And then one day we had a very strong clash, which was the day that I kicked the gate . . . I broke the gate. I said, ‘Now no one is going to be locked up anymore, and I am leaving, I’d like to see you try to hold me here!’

Tamires left that workplace and continued to work in sex work in less restrictive, but still not always ideal, settings.

These stories vividly illustrate problems with the portrayal of ‘victims of trafficking’ as passive, non-agential objects in dominant anti-trafficking discourse. Furthermore, while it is clear that both Tamires and Isabelle were victims of crime(s), and that it would be in their interests for the law to protect them from such victimisation, just as the law should protect every person from false imprisonment, rape, assault, and so on, their stories also highlight problems with the kinds of interventions spawned by anti-trafficking discourse. When sex work is framed as slavery, and/or when crimes such as these are bundled together under the heading of ‘sex trafficking’ and imagined as ineluctably connected to prostitution, the goal of such interventions is not to enforce the rights of women in sex work as persons, and certainly not to champion and enforce their rights as workers. Rather, it is to remove them from sex work and prevent them from being ‘re-enslaved’ by putting obstacles in the way of sex commerce per se. Such measures would be extremely unwelcome to our interviewees, including Isabelle and Tamires, as they would shut down the one avenue to social mobility open to them.

However, our data also show why the dominant discourse cannot be countered simply by insisting that ‘not all sex workers are victims of trafficking’. Tamires and Isabelle’s stories reveal that over time, the same individuals can be positioned ‘as both victims of sex trafficking and voluntary sex workers, depending on the point at which their situations come to be known and documented – by researchers, police, NGOs or immigration authorities (or indeed by researchers)’ (Yea, 2012: 55). Moreover, the fact that they escaped an abusive situation does not mean they will never again find themselves ‘in a jam’, as Isabelle put it. Although currently free of these types of restraints, they, and our other research participants, could in future potentially run into similar difficulties.

Moreover, the preoccupation with choice, whether expressed through anti-trafficking campaigners’ concern with private actors’ use of force and coercion in sex work, or ‘sex positive’ sex worker rights activists’ concern with bodily autonomy and sexual liberation, deflects attention from the structural factors that compel men, women, and children to decide between options that are not of their choosing under the social relations of global capitalism. Without social protections *against* the market and in the absence of any alternative means of attaining a modicum of economic security and ‘making progress’ with life plans, consent to any given form of work does not equate to freedom (O’Connell and Davidson, 2014: 524). As the data presented in this article show, by focusing on the trajectories of sex working women’s lives over time, it is possible to better grasp both the protean nature of sex work, and the impossibility of fixing people’s participation in it as either forced or free.
